# Detection and Characterization of Web-Based Pediatric COVID-19 Vaccine Discussions and Racial and Ethnic Minority Topics: Retrospective Analysis of Twitter Data

**DOI:** 10.2196/48004

**Published:** 2023-11-30

**Authors:** Tiana McMann, Christine Wenzel, Nicolette Le, Zhuoran Li, Qing Xu, Raphael E Cuomo, Tim Mackey

**Affiliations:** 1 Global Health Program, Department of Anthropology, University of California, San Diego, La Jolla, CA, United States; 2 Global Health Policy and Data Institute, San Diego, CA, United States; 3 S-3 Research, San Diego, CA, United States; 4 Department of Anesthesiology, School of Medicine, University of California, San Diego, San Diego, CA, United States

**Keywords:** COVID-19 vaccine, vaccine hesitancy, pediatric vaccine, pediatric COVID-19 vaccine, vaccine beliefs, vaccine-related concerns, vaccine-related confidence, vaccine barriers, vaccine facilitators, racial and ethnic minority

## Abstract

**Background:**

Despite pediatric populations representing a smaller proportion of COVID-19 cases and having a less severe prognosis, those belonging to racial and ethnic minority groups are at an increased risk of developing more severe COVID-19–related outcomes. Vaccine coverage is crucial to pandemic mitigation efforts, yet since the start of the COVID-19 pandemic, vaccine hesitancy has increased and routine pediatric immunizations have decreased. Limited research exists on how vaccine hesitancy may contribute to low pediatric COVID-19 vaccine uptake among racial and ethnic minority populations.

**Objective:**

This study aimed to characterize COVID-19 vaccine–related discussion and sentiment among Twitter users, particularly among racial and ethnic minority users.

**Methods:**

We used the Twitter application programming interface to collect tweets and replies. Tweets were selected by filtering for keywords associated with COVID-19 vaccines and pediatric-related terms. From this corpus of tweets, we used the Biterm Topic Model to output topics and examined the top 200 retweeted tweets that were coded for pediatric COVID-19 vaccine relevance. Relevant tweets were analyzed using an inductive coding approach to characterize pediatric COVID-19 vaccine–related themes. Replies to relevant tweets were collected and coded. User metadata were assessed for self-reporting of race or ethnic group affiliation and verified account status.

**Results:**

A total of 863,007 tweets were collected from October 2020 to October 2021. After outputting Biterm Topic Model topics and reviewing the 200 most retweeted tweets, 208,666 tweets and 3905 replies were identified as being pediatric COVID-19 vaccine related. The majority (150,262/208,666, 72.01%) of tweets expressed vaccine-related concerns. Among tweets discussing vaccine confidence, user replies expressing agreement were significantly outweighed by those expressing disagreement (1016/3106, 32.71% vs 2090/3106, 67.29%; *P*<.001). The main themes identified in the Twitter interactions were conversations regarding vaccine-related concerns including adverse side effects, concerns that the vaccine is experimental or needs more testing and should not be tested on pediatric populations, the perception that the vaccine is unnecessary given the perceived low risk of pediatric infection, and conversations associated with vaccine-related confidence (ie, the vaccine is protective). Among signal tweets and replies, we identified 418 users who self-identified as a racial minority individual and 40 who self-identified as an ethnic minority individual. Among the subcodes identified in this study, the vaccine being protective was the most discussed topic by racial and ethnic minority groups (305/444, 68.7%).

**Conclusions:**

Vaccine-related concerns can have negative consequences on vaccine uptake and participation in vaccine-related clinical trials. This can impact the uptake and development of safe and effective vaccines, especially among racial and ethnic minority populations.

## Introduction

COVID-19 has caused significant morbidity and mortality globally, leading to over 6 million hospitalizations and claiming more than 1 million lives in the United States alone as of September 2023 [1,2]. Specific risk factors for clinical severity such as older age, underlying medical conditions, and racial and ethnic minority status have been previously identified [3]. Although all age groups can be infected by COVID-19, children represent a smaller proportion of all cases reported and generally present with milder symptomology and improved clinical outcomes when compared to adults [4,5]. Despite the overall lower risk, the American Academy of Pediatrics reported that there have been over 15.6 million COVID-19 cases in children reported in the United States as of May 2023, and since the start of the pandemic, incidence among pediatric populations plateaued at an average of approximately 24,000 cases per week and have more recently declined [6,7].

Furthermore, similar risk factors for severe COVID-19 infections identified in adults, such as racial and ethnic minority status, also place pediatric populations at increased risk, whereas other serious conditions, such as multisystem inflammatory syndrome in children, represent a unique health risk in this group [4,8]. Additionally, COVID-19 pediatric hospitalization rates, although lower than those of adults, mimic rates of prevaccine hospitalizations of now vaccine-preventable diseases [9]. However, pediatric COVID-19 hospitalization rates are not uniformly distributed, with multiple studies identifying higher rates and intensive care unit admissions among Hispanic or Latino and non-Hispanic or non-Latino Black children [10,11].

Crucially, parental uncertainty toward the pediatric COVID-19 vaccines has and continues to be a key concern and is a driving factor in the success or failure of vaccination programs and achieving high immunization rates. Globally, vaccine hesitancy rates vary by characteristics and predictors, with parents and youth in some countries expressing low vaccine hesitancy and high vaccine confidence, whereas others express negative vaccine sentiment and outright refusal [12-14]. Reflecting these conflicting attitudes and opinions, as measured in June 2022, more than 18 million children in the United States had yet to receive their first dose of a COVID-19 vaccine [15]. Furthermore, differential rates of vaccination in pediatric populations mimic those of adults, with racial and ethnic minority populations historically having lower COVID-19 vaccine uptake [16,17].

Prior to the COVID-19 pandemic, barriers to increased adolescent immunization rates included parental acceptance of vaccines, vaccine knowledge, and attitudes toward vaccination [18]. Prepandemic conditions included parents outright refusing and others choosing to delay or spread out routine vaccinations [19]. Since the start of the pandemic, vaccine hesitancy, especially concerning pediatric vaccinations, has increased, and overall pediatric vaccination rates have declined [20,21]. Similar to vaccine uptake, vaccine hesitancy is not uniformly distributed, with greater hesitancy existing among African American and Hispanic populations [15,22,23]. Although pediatric mortality has declined by 96% to 100% in the United States due to recommended routine vaccinations, recent vaccine hesitancy has contributed to outbreaks of previously vaccine-preventable diseases such as measles and influenza, emphasizing the importance of countering misinformation and overall hesitancy sentiment [22].

Recent pediatric COVID-19 vaccine research has largely focused on the efficacy and safety of the vaccine, with few papers examining individuals’ or communities’ opinions on vaccine administration [24-26]. Furthermore, the limited but growing research on racial and ethnic minority COVID-19 vaccine hesitancy has focused primarily on adult populations, although some studies have reported varying parental intent to vaccinate children [14,27]. However, no study to our knowledge has focused on pediatric COVID-19 barriers and facilitators among racial and ethnic minority groups and the extent of web-based engagement generated by information sharing and measured the impact of certain concerns and beliefs within these populations. Hence, additional research is needed to better characterize knowledge, attitudes, and behaviors associated with pediatric COVID-19 vaccines among disproportionately impacted groups, namely racial and ethnic minority populations.

The first step to tailored outreach efforts is to increase understanding of the barriers and concerns held by disproportionately affected and historically underrepresented groups and determine whether these beliefs are representative within a particular community. Social media’s emergence as a popular channel for information seeking and sharing and health behavior discussion has resulted in several studies characterizing COVID-19 and vaccine confidence and hesitancy [28,29]. Hence, the aim of this study was to add to this body of literature using approaches in natural language processing and content analysis to identify and characterize pediatric vaccine discussion topics, sentiment, and user interactions, including among users self-reporting racial and ethnic minority affiliation on Twitter (now rebranded as “X”), a common microblogging social media platform used by 1 in 5 US adults [30].

## Methods

### Overview

**Figure 1.**
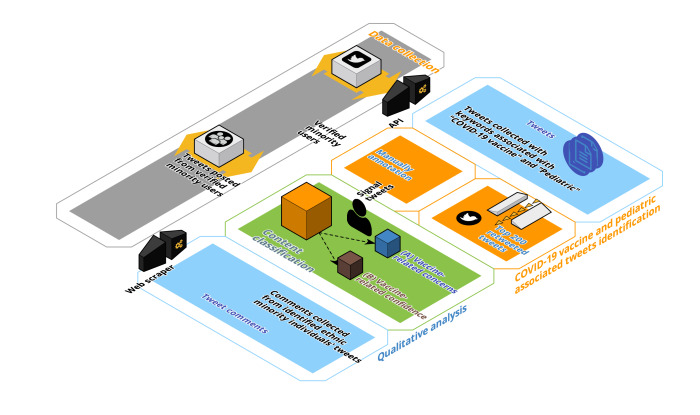
Methodology summary and flowchart. The general methodology of the study is broken down into (1) data collection using the Twitter API; (2) COVID-19 and pediatric keyword filtering; (3) Biterm Topic Model and tweet selection; (4) qualitative analysis; and (5) metadata analysis. API: application programming interface.

This study was conducted in three distinct phases: (1) data collection of COVID-19 vaccine– and pediatric-related tweets using keyword querying and filtering; (2) using unsupervised machine learning with topic modeling to identify topics and themes relevant to vaccine confidence, hesitancy, and minority user topics specific to COVID-19 pediatric vaccination; and (3) conducting in-depth qualitative analysis of tweets and comments using an inductive coding approach. Additionally, user profile metadata from all publicly available tweets were collected to assess if users self-reported racial or ethnic minority affiliation and whether they had verified Twitter accounts. A visual summary of the study methodology is provided in Figure 1.

### Ethical Considerations

As this study only analyzed secondary, publicly available data and does not report any individually identifiable information on users, it was deemed exempt by WCG IRB.

### Data Collection

We first manually searched for tweets with COVID-19 vaccine–related keywords on Twitter, the social media platform selected for this study. After assessing the returned results, we generated a list of keywords and hashtags that are commonly used in COVID-19 vaccine Twitter discussions, such as “Moderna,” “COVID19 vaccine,” and “Pfizer” (see Table 1 for the full list of study keywords). Data collection was conducted from October 24, 2020, to October 1, 2021, using the Twitter public application programming interface (API) to prospectively collect tweets that contained study keywords. Two sets of streaming data were collected; 1 set contained only original tweets (non-retweeted posts), and the other set contained only retweets. With the same manual search process, we generated keywords for general pediatric topics, which are “pediatric” and “paediatric,” and filtered our general COVID-19 data sets for tweets that contained these 2 keywords to generate a separate filtered non-retweeted and retweeted data set. From both non-retweeted and retweeted data sets, we collected all associated comments generated by users replying to these tweets with the full-archive Twitter API using the “Conversation_id” attribute to better sample both original tweets and their interactions (ie, comments) with these keywords.

**Table 1.** The keywords related to “COVID-19 vaccine” and “pediatric COVID-19 vaccine” that were selected in this study.

Topic	Related keywords
COVID-19 vaccine	Sputnik V, Gam-COVID-Vac, Moderna vaccine, mRNA-1273, AXD1222, COVID19 vaccine, ChAdOx1, Pfizer, BioNTech, Johnson vaccine, AstraZeneca, J&J’s vaccine, JNJ-78436735, Ad26.COV2.S, AZD1222, Oxford vaccine, and Comirnaty
Pediatric COVID-19 vaccine	Pediatric and paediatric

### Unsupervised Machine Learning

Due to the large volume of data for the non-retweeted pediatric data set, we used natural language processing and unsupervised machine learning to extract topics of interest and the corresponding tweets relevant to our study objectives. We used the Biterm Topic Model (BTM), an unsupervised machine learning approach that cluster texts into different topics and outputs the word terms that are the most correlated to each topic. We chose the BTM as it is efficient in analyzing short texts (such as tweets that are limited to 280 characters) and due to its use in prior health and COVID-19 topic exploration studies, particularly when existing training data for supervised machine learning approaches are not available [31-33]. Because the non-retweeted pediatric data set volume was double the size of usual BTM training sets used in prior studies, we split the non-retweeted pediatric data set into 2 even data sets for BTM topic modeling output. For each BTM process, we used the parameter *k*=20, which generated 20 topics for each set of BTM modeling phases. For each topic, we outputted the top relevant terms for each topic and ranked all tweets within that topic and then outputted the 10 most retweeted tweets (based on retweet counts) that were the most correlated within the outputted topic for purposes of further human review and annotation.

### Content and Statistical Analysis

This study’s manual content analysis of tweets focused on characterizing specific COVID-19 pediatric vaccine–related discussion and sentiment, specifically among racial and ethnic minority groups. Following the use of BTM, the top 10 retweeted tweets from each topic cluster were outputted and coded using a binary coding scheme to identify tweets relevant to the topic of pediatric COVID-19 vaccines (ie, “signal”). Tweets were deemed as signal tweets if they (1) appeared to be user generated (ie, not posted by organizations or news or media outlets) and (2) discussed a topic relevant to the pediatric COVID-19 vaccine, including its indication, safety, efficacy, approval or authorization, beliefs, and associated barriers and facilitators. Topic clusters that had retweeted tweets that did not meet the study objective were excluded from further analysis (ie, “noise”). Tweets related solely to news or media coverage about the vaccine, advertisements, and tweets not related to the pediatric COVID-19 vaccine (eg, tweets associated with pediatric vaccines for other diseases) were considered noise. All tweets were first reviewed by the first (TM), second (CW), and third (NL) authors independently, and a general inductive approach was then used to develop a coding framework for tweets to assess thematic content.

Following the initial binary coding scheme and review of all tweets, all detected themes were inductively classified into 2 parent codes: vaccine-related confidence and vaccine-related concerns. Subcodes were inductively added to the codebook under the 2 parent codes. These attributes were selected by the first 3 authors, with high interrater reliability (Cohen *κ*=0.95). Discrepancies were resolved through discussion among the first through third authors. Following the qualitative content analysis of the top retweeted tweets, all comments to signal tweets were retrieved, and the first 3 authors followed a binary coding scheme for the relevance of the comment to the original tweet. Comments were also manually annotated for agreement, disagreement, or neutral sentiment toward the original tweet. All comments were coded independently and achieved high intercoder reliability (Cohen *κ*=0.95).

A 2-by-2 table for user replies was constructed. The exposure condition was a parent tweet (ie, by parent tweet, we mean a tweet that was selected for this study and generated additional user interactions through comments) expressing confidence in the pediatric vaccine, with the counterfactual being a parent tweet expressing concern; the outcome condition was a user reply expressing either agreement or disagreement with the parent tweet. A *χ*^2^ test was performed to determine if the proportion agreeing with confidence-expressing tweets was significantly different than the proportion disagreeing with confidence-expressing tweets. Statistical analysis was performed in RStudio (version 3.6.1; Posit). A *P* value of <.05 was considered statistically significant.

### User Metadata Analysis

We wanted to further characterize specific topics, discussions, and sentiments associated with pediatric COVID-19 vaccines that were specific to racial and ethnic minority populations. We examined publicly available metadata of users associated with signal tweets and signal comments for self-identifiable minority status as well as Twitter verified user account status. In this study, we included 4 major racial groups (American Indian and Alaska Native, Asian, Black or African American, and Native Hawaiian or Other Pacific Islander) and 1 ethnic group (Hispanic or Latino). The classification used only publicly available profile data from the users’ profile on Twitter to assess whether there was sufficient information to identify at least 1 of the abovementioned minority groups. If a user included no self-identification information within their public profile bio, no racial or ethnic minority status was assumed. These data were collected for purposes of aggregation, and no results contained in this study include individually identifiable information or make any representation to the accuracy of a claimed minority or ethnic classification of a user.

## Results

### Collected Data

We collected a total of 863,007 tweets from Twitter over the approximately 1-year study period, which were filtered for COVID-19– and pediatric-related keywords. After applying the BTM, we reviewed the top 200 most retweeted tweets (representing 233,612 tweets and retweets) from each topic cluster output, from which 163 (81.5%) of the 200 most retweeted tweets were identified as signal tweets based on our binary coding approach. These signal tweets corresponded to a total of 208,666 tweets and retweets (208,666/863,007, 24.18% of the entire corpus) and specifically included user-generated topics related to the pediatric COVID-19 vaccine. From this set of tweets, a total of 15,524 user replies via comments were collected. Within these user replies, 6224 replies were posted in response to tweets from verified racial or ethnic minority users that were then selected for further racial and ethnic minority content–specific analysis. Of the 6224 comment replies selected for analysis, 3905 (62.74%) were relevant to the parent tweet’s COVID-19 vaccine topic and were further analyzed for agreement, disagreement, or neutral sentiment.

### Content and Statistical Analysis

Based on our qualitative analysis and inductive coding approach of tweets and retweets, we derived 8 topics within 2 major parent domains (refer to Table 2 for identified topic themes and anonymized and paraphrased examples from tweets). The detected topics were first classified into 2 major domains: vaccine-related concerns (150,262/208,666, 72.01%) and vaccine-related confidence (58,404/208,666, 27.99%). Of the 3905 comment replies to the parent tweets that were identified as signal retweets using our binary coding approach, 3385 (86.68%) were in response to vaccine-related confidence tweets and 520 (13.32%) were in response to vaccine-related concern tweets. Of 3385 vaccine-related confidence reply comments, we found that 1016 (30.01%) users agreed, 2090 (61.74%) disagreed, and 279 (8.24%) had neutral sentiment toward conversations regarding the vaccine being safe and protective. In response to vaccine-related concerns, 278 (53.5%) out of 520 users agreed, 219 (42.1%) disagreed, and 23 (4.4%) had neutral sentiment toward conversations regarding the vaccine having adverse side effects, it being too experimental, and other vaccine concern topics.

**Table 2.** Code list and identified topic themes (including deidentified and paraphrased examples). Specific company names have been deidentified and replaced with generic labels (eg, “Company A”).

Topic and code number		Code name	Example	Tweets and retweets (n=208,666), n (%)
**A. Vaccine-related concerns**				
	A-1	Adverse side effects	Boston Children’s Hosp series of 15 post Pfizer C19 vax myocarditis cases revealed 80% had “late gadolinium enhancement,” a prognostic marker assoc with increased risk (~4.6 fold) for adverse cardiac events long term.	37,571 (18.01%)
	A-2	Requires more testing (experimental, unethical, and questioning approval)	Healthy children do not need this vaccine and it’s been advised not to give them the jab. This won’t keep a single school open or save children’s lives. This is immoral, unethical and, what’s worse, you KNOW it.	40,279 (19.3%)
	A-3	Control tactic	The Johnson regime is intent on ruining the country and destroying freedom. They have announced vaccine passports and experimental injections for children and now a #Lockdown. It is time for regime change.	12,122 (5.81%)
	A-4	Questioning authority	Why is Biden telling children to get the vaccine. He is not a medical doctor. This girl should be allowed to sue Biden for practicing medicine without a license.	8420 (4.04%)
	A-5	Vaccine is unnecessary (high risk to individual benefit)	J&J announced plans to test the shot on newborns, despite the risks and evidence that COVID poses nearly no risk to healthy children.	36,859 (17.66%)
	A-6	Company history	In 1996, one of Pfizer’s drugs was still in clinical stage of development when it was tested on about 200 children without consent. Pfizer claimed it was “safe,” but 181 kids were injured and 11 died.	15,011 (7.19%)
**B. Vaccine-related confidence**				
	B-1	Vaccine is protective	What amazing news to wake up to! Pfizer vaccine provided 100% protection in 12-15 y olds. The sample is small, but gives me so much hope for schools opening soon.	35,975 (17.24%)
	B-2	Vaccine is safe	JUST IN - Pfizer has started late-stage clinical trials of their #COVID19 vaccine in young children ages 5 to 11	22,429 (10.75%)

Within the vaccine-related concern parent topic (parent code A), we identified tweets that shared concerns (A-1) regarding possible adverse side effects of the pediatric COVID-19 vaccine; (A-2) that the vaccine required more testing; (A-3) that the vaccine was being used as a control tactic; (A-4) questioning authority figures associated with vaccination or government (eg, the perception that current President Joe Biden is wrongly encouraging vaccination and a statement expressing the idea that certain government authorities lack the requisite expertise to demand health initiatives); (A-5) that the vaccine was unnecessary; and (A-6) about the history or purported activities of pharmaceutical companies (eg, previous mismanagement and greed claims against pharmaceuticals). Within the vaccine-related confidence parent topic (parent code B), we identified tweets that shared that the pediatric COVID-19 vaccine is (B-1) protective and (B-2) safe (see Table 2). Among these topics, tweets expressing concerns regarding the vaccine requiring more testing and that it was still experimental or should not be approved had the highest volume (A-2; 40,279/208,666, 19.3%), followed by discussion surrounding the possible adverse side effects associated with the pediatric COVID-19 vaccine (A-1; 37,571/208,666, 18.01%).

Among those who responded to tweets via comments expressing confidence in pediatric vaccines, 30.01% (1016/3385) agreed, 8.24% (279/3385) were neutral, and 61.74% (2090/3385) disagreed. Among those who responded to tweets expressing concern about pediatric vaccines, 53.5% (278/520) agreed, 4.4% (23/520) were neutral, and 42.1% (219/520) disagreed. Agreement with tweets was determined by (1) explicit agreement statements (eg, “I agree,” “true,” “definitely,” etc); (2) supportive statements that reiterate or add to the original parent tweet (eg, “this is great information”); (3) personal, supportive anecdotes (eg, “this has happened to me”); or (4) contextual information on a case-by-case basis such as the use of supportive emojis within a reply (eg, thumbs up). Disagreement with tweets was determined by (1) explicit disagreement statements (eg, “I disagree,” “this is not true,” etc); (2) counterarguments or sharing counterfactual news or informational links; (3) criticizing the tweet or the author of the tweet (eg, “how could you post this fake information,” etc); or (4) contextual information on a case-by-case basis such as the use of opposing emojis within a reply (eg, thumbs down). Neutrality was determined by (1) requesting additional information or (2) the lack of strong agreement or disagreement language.

Excluding neutral-sentiment responses, the proportion of user replies expressing agreement with provaccine (ie, confidence) tweets was significantly lower than those expressing disagreement with provaccine tweets (1016/3106, 32.71% vs 2090/3106, 67.29%; *χ*^2^_1_=98.6, *P*<.001). As tweets in this sample were either provaccine or antivaccine (ie, concern), statistical testing also indicated that the proportion expressing agreement with antivaccine tweets was significantly higher than those expressing disagreement with antivaccine tweets (278/497, 55.9% vs 219/497, 44.1%; *χ*^2^_1_=98.6, *P*<.001).

### User Metadata Analysis

In total, this study identified 418 users who self-identified as a racial minority individual and 40 users who self-identified as an ethnic minority individual among signal parent tweets and reply comments. From the top 200 retweeted tweets included for analysis in this study, 14 were identified as being posted by a verified racial or ethnic minority Twitter user, who generated 2.8% (24,140/863,007) of the tweets or retweets of the entire corpus. Specifically, from the sample of identified verified racial or ethnic minority Twitter users, 3905 replies to these tweets were identified as a signal tweet, of which 444 (11.37%) were identified as being posted by a user who also self-identified with a racial or ethnic minority group, whereas 673 (17.23%) were from White users and 2788 (71.4%) were unable to be identified. We identified that of the 3905 users who replied to verified Twitter accounts, 9 (0.23%) self-identified as American Indian or Alaskan Native, 310 (7.94%) as Asian, 84 (2.15%) as Black or African American, 39 (1%) as Hispanic or Latino, and 2 (0.05%) as Native Hawaiian or Other Pacific Islander. Within the parent topic of vaccine-related confidence, 331 racial minority individuals and 37 ethnic minority individuals posted reply tweets; within the parent topic of vaccine-related concern, 74 racial minority individuals and 2 ethnic minority individuals posted reply tweets.

Among the 8 subcodes identified in this study, the vaccine being protective (B-1) was the most discussed topic by racial and ethnic minority groups (305/444, 68.7%). Additionally, 287 reply comments were posted by users who self-identified as a racial minority individual and 18 were by those who self-identified as an ethnic minority individual. Overall, 6 American Indian or Alaskan Native, 243 Asian, 37 Black or African American, 18 Hispanic or Latino, and 1 Native Hawaiian or Pacific Islander users replied to tweets that discussed the vaccine as being protective. Importantly, a larger proportion of reply comments disagreed with provaccine parent tweets, with Asian populations specifically representing most of the identified users with disagreement sentiment (170/261, 65.1%; refer to Tables 3 and 4 for a complete breakdown of racial and ethnic minority sentiment for topics related to vaccine-related concerns and confidence). Among self-identified Black or African American populations in this study, the vaccine being safe (B-2) and the adverse effects of the vaccine (A-1) were tied for the topics receiving the second most engagement among these groups, with 23 reply comments in each of these topics. Finally, the vaccine being unnecessary for pediatric populations (A-5) was the topic with the second most engagement among Asian populations. Furthermore, contradicting the initial finding of disagreement with vaccine-related confidence was the disagreement of vaccine-related concerns among 61% (30/49) of comment replies posted by Asian users.

**Table 3.** Vaccine-related concern sentiment by racial and ethnic minority status.

Race or ethnicity	Agree, n (%)	Disagree, n (%)	Neutral, n (%)
American Indian or Alaska Native (n=1)	0 (0)	1 (100)	0 (0)
Asian (n=49)	17 (35)	30 (61)	2 (4)
Black or African American (n=24)	11 (46)	12 (50)	1 (4)
Hispanic or Latino (n=2)	1 (50)	1 (50)	0 (0)
Native Hawaiian or Other Pacific Islander (n=0)	0 (0)	0 (0)	0 (0)

**Table 4.** Vaccine-related confidence sentiment by racial and ethnic minority status.

Race or ethnicity	Agree, n (%)	Disagree, n (%)	Neutral, n (%)
American Indian or Alaska Native (n=8)	3 (37.5)	5 (62.5)	0 (0)
Asian (n=261)	75 (28.7)	170 (65.1)	16 (6.1)
Black or African American (n=60)	26 (43.3)	26 (43.3)	8 (13.3)
Hispanic or Latino (n=37)	15 (40.5)	20 (54.1)	2 (5.4)
Native Hawaiian or Other Pacific Islander (n=2)	0 (0)	1 (50)	1 (50)

## Discussion

### Principal Findings

This study found 208,666 signal tweets related to pediatric COVID-19 vaccine topics and 3905 signal comments in response to relevant parent tweets. The tweets included 2 parent categories of vaccine-related concern and vaccine-related confidence topics and 8 corresponding subcodes. In relation to conversations (tweets) and interactions (comment replies) from racial or ethnic minority users on Twitter, we found 458 tweets and replies posted by users who self-identified as a member of 1 of 4 racial minority groups or 1 ethnic minority group.

For all tweets reviewed, we found that close to three-quarters (150,262/208,666, 72.01%) of all discussions reviewed expressed vaccine-related concerns, with the subtopic of the pediatric COVID-19 vaccine requiring more testing (A-2) driving most of the conversations on Twitter (40,279/150,262, 26.81%). We also found that most user replies reviewed were in response to vaccine-related confidence tweets (ie, that the vaccine was safe and protective; 3385/3905, 86.68%), although the majority (2090/3385, 61.74%) of these replies disagreed with this sentiment of supporting vaccination. Additionally, the proportion expressing agreement with provaccine tweets was significantly lower than those expressing disagreement with provaccine tweets (1016/3106, 32.71% vs 2090/3106, 67.29%; *P*<.001). These findings may indicate that when provaccine sentiment is shared on Twitter, a larger proportion of interactions ensuing may conversely generate antivaccine sentiment from users in the form of comment replies, which is a concerning finding particularly as over 800 users in this study self-identified as belonging to a racial or ethnic minority group and may have been exposed to predominantly negative vaccine sentiment content.

Among users self-reporting their race or ethnic status in response to a verified minority user Twitter account, Asian, Black or African American, and Hispanic or Latino groups were the top 3 reported affiliations, with other racial groups (American Indian or Alaskan Native and Native Hawaiian or Other Pacific Islander) having a lower number of users relative to their smaller proportion of the US population. Most racial or ethnic minority users’ comment replies were in response to vaccine confidence, specifically, the vaccine being protective, with generally more of these minority users disagreeing with the vaccine-related confidence sentiment but conversely also more users disagreeing in replies to the vaccine-related concern sentiment. This may indicate that different and specific minority groups on Twitter are having separate conversations and interactions regarding vaccine confidence or hesitancy, with some pushing back against vaccine-related concerns (such as Asian users) and some disagreeing with vaccine confidence statements (again with Asian users, although stratification for different Asian ethic subgroups may yield more specific results). In contrast, Black or African American users were more evenly split on their sentiment toward vaccine-related confidence or concern in their replies to tweets.

Specific Twitter verified user accounts reporting racial or ethnic minority affiliation may have also influenced these conversation topic groupings, which included users who are political figures, epidemiologists, and prominent journalists who have high follower accounts (range 56,453-22,474,858). This high number of Twitter followers in turn generated a higher volume of interactions via user replies. Topics in the tweets of these verified users varied, including mistrust regarding federal regulatory agencies, general COVID-19 vaccine announcements, and tweets related to vaccine or COVID-19 fear-mongering language.

Overall, clinical trial results supporting vaccine authorization, as with other aspects of the COVID-19 pandemic, was met with mixed sentiments [34,35]. Public opinion about the need for a pediatric vaccine varied, with individuals, primarily those who are parents, questioning whether COVID-19 posed enough risk to children to necessitate the testing and development of a pediatric vaccine [36]. The results of our study reinforce these observations, similarly finding that public opinion on Twitter toward vaccine confidence- and concern-related tweets and interactions is mixed, including when specifically examining racial and ethnic minority user sentiment.

### Limitations

This study has certain limitations. First, the primary aim of the study was to characterize pediatric COVID-19 vaccine discussion and attitudes among general users as well as racial and ethnic minority users on Twitter. By using a single social media platform, our scope is limited based on the demographic of Twitter users and may not be representative of general attitudes among various racial and ethnic minority groups. Additionally, we only collected data from Twitter and limited our study keywords to the English language. This likely biased study results to native English speakers, excluding minority individuals for whom English is a second language or those who do not speak English, thus further limiting generalizability. Additionally, our keywords related to COVID-19 vaccine topics were chosen based on our own manual searches on the platform but may not have been inclusive of all conversations related to the study aims. Finally, we identified users’ race and ethnicity status based solely on users’ publicly available metadata, and we did not cross-validate the veracity of users’ race and ethnicity status with any other sources or follow-up. Future studies should explore combining multiple data layers from different sources to better validate users’ race and ethnicity status as well as to better assess if users’ reported attitudes, perceptions, and behavior are associated with validated vaccine confidence, hesitancy, or uptake using other data sources.

### Conclusions

Although the results from this study are primarily exploratory, they nevertheless provide important and diverse insights on current beliefs, attitudes, opinions, and possible behavior queues toward intent, willingness, and hesitancy to vaccinate children to protect them from COVID-19. Hence, our study results have the potential to inform the design of future health education, vaccination campaigns, and other health promotion efforts that can be targeted toward specific minority users on the web while also identifying relevant themes and topics that can influence vaccine confidence and hesitancy sentiment. Incorporating social listening with traditional public health surveillance approaches is critical to ensuring equitable vaccine uptake, including in the context of new vaccine candidates and COVID-19 boosters among both parents and their children. Future studies may look to further stratify racial and ethnic subgroups to better understand how intraracial attitudes and behaviors may vary to more effectively target pediatric vaccine campaigns.

## Abbreviations

API: application programming interface
BTM: Biterm Topic Model
